# Feasibility and Safety of Robotic Para-Aortic Lymphadenectomy Using the hinotori™ Surgical Robot System: A First-in-Human Experience

**DOI:** 10.7759/cureus.103434

**Published:** 2026-02-11

**Authors:** Seiji Mabuchi, Tomoyuki Sasano, Tomoko Ueda, Yu Wakimoto, Hiroshi Tsubamoto

**Affiliations:** 1 Department of Obstetrics and Gynecology, Hyogo Medical University, Nishinomiya, JPN

**Keywords:** abdominal aorta, endometrial neoplasms, lymph node excision, ovarian neoplasms, para-aortic lymphadenectomy, robotic surgical procedures

## Abstract

This case series reports the first cases of para-aortic lymphadenectomy performed using the hinotori™ Surgical Robot System (Medicaroid Corporation, Kobe, Japan), Japan’s first domestically developed robotic surgical platform. Three patients with gynecologic malignancies (two endometrial and one ovarian cancer) underwent curative surgery, including total hysterectomy, bilateral salpingo-oophorectomy, pelvic lymphadenectomy, and para-aortic lymphadenectomy. All procedures were completed robotically without conversion or intraoperative complications. Operative time ranged from 461 to 512 minutes, and blood loss from 10 to 50 mL. A total of 9-38 para-aortic lymph nodes (median 26) were retrieved, comparable to yields from laparoscopic or da Vinci®-assisted procedures, confirming oncologic adequacy. Postoperative recovery was uneventful in all cases. These cases demonstrate that para-aortic lymphadenectomy using the hinotori™ system is technically feasible and safe. Expanding its use from well-established pelvic surgery to advanced oncologic procedures marks an important milestone for robotic surgery in Japan. Further studies are needed to refine protocols and assess outcomes.

## Introduction

Para-aortic lymphadenectomy (PALND) plays a crucial role in the surgical staging and therapeutic decision-making of gynecologic malignancies, where para-aortic lymph node metastasis is a key prognostic factor [1]. While its definitive survival benefit remains under investigation, several randomized controlled trials (RCTs) are ongoing to determine its therapeutic impact [2-4]. Traditionally, this procedure was performed via laparotomy, which is associated with significant surgical trauma and delayed postoperative recovery due to the large abdominal incision. With increasing patient demand for minimally invasive options and rapid technological advances, minimally invasive approaches, including laparoscopy and robot-assisted surgery, have gained popularity [5,6]. Minimally invasive PALND requires a high level of technical skill and experience, as the procedure is performed within a narrow operative field adjacent to major vessels, including the inferior vena cava, aorta, and renal vessels, and carries potential risks of vascular, uterine, and intestinal injury. In this context, robot-assisted surgery may offer advantages over open surgery, including enhanced three-dimensional visualization, improved ergonomics, and tremor filtration, potentially enabling safer and more precise dissection in a narrow retroperitoneal space. However, to date, all reported cases of robot-assisted PALND have been performed using the da Vinci® system [5,6].

The hinotori™ Surgical Robot System is a novel robotic-assisted surgical platform developed in Japan by Medicaroid Corporation (Kobe, Japan), a joint venture between Kawasaki Heavy Industries and Sysmex Corporation. It became the first domestically developed robotic system to gain regulatory approval in Japan in August 2020. The system features a surgeon console and a patient-side cart with four robotic arms, each with eight degrees of freedom, enhancing flexibility and reducing arm collision. Its software-based pivot calibration and ergonomic design aim to optimize performance and surgeon comfort [7]. Clinical use began in 2020 in urology [8], later expanding to gastrointestinal, thoracic, and gynecologic surgeries [9]. In gynecology, it has mainly been applied to endopelvic surgeries for benign uterine diseases and early-stage endometrial cancer [10]. Importantly, PALND using the hinotori™ system has never been reported in any surgical specialty.

Here, we present the world’s first cases of PALND performed using the hinotori™ system in patients with endometrial or ovarian cancer, evaluating its safety, feasibility, and perioperative outcomes, in the context of previously published da Vinci®-based experiences.

## Case presentation

Surgical procedures

Patient and Ethical Considerations

Three patients with gynecologic malignancies requiring full staging surgery, including PALND, were enrolled. All provided written informed consent. Procedures were approved by the High-Level New Medical Technology Evaluation Committee of Hyogo Medical University Hospital and performed as self-financed medical care. The Institutional Review Board waived the requirement for approval, as a case series of three patients did not meet the definition of human-subject research.

Instrument Placement

**Figure 1:**
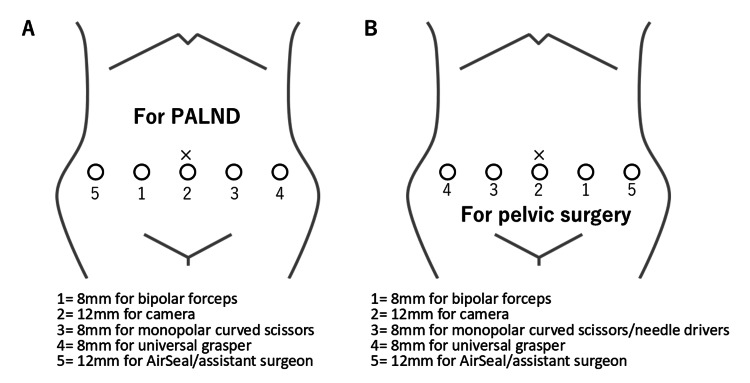
Positioning of the trocars. (A) Trocar placement for para-aortic lymphadenectomy (PALND). (B) Trocar placement for pelvic surgery. Original illustration created by the authors.

Trocar placement for PALND is shown in Figure 1A. The abdomen was entered via the open technique, and a 12-mm balloon trocar was placed infraumbilically. Three 8-mm robotic trocars were positioned horizontally below the umbilicus, and an additional 8-mm assistant port was placed in the lower quadrant to maintain pneumoperitoneum at 12 mmHg using an AirSeal® system (CONMED Corporation, Largo, FL). The patient was placed in a 20° Trendelenburg. Small bowel and omentum were retracted cranially before docking. The hinotori™ cart was docked from the right for PALND, and then undocked and rotated for pelvic procedures (Figure 1B). Perioperative prophylaxis included intravenous flomoxef and low-molecular-weight heparin.

Surgical Technique

**Figure 2:**
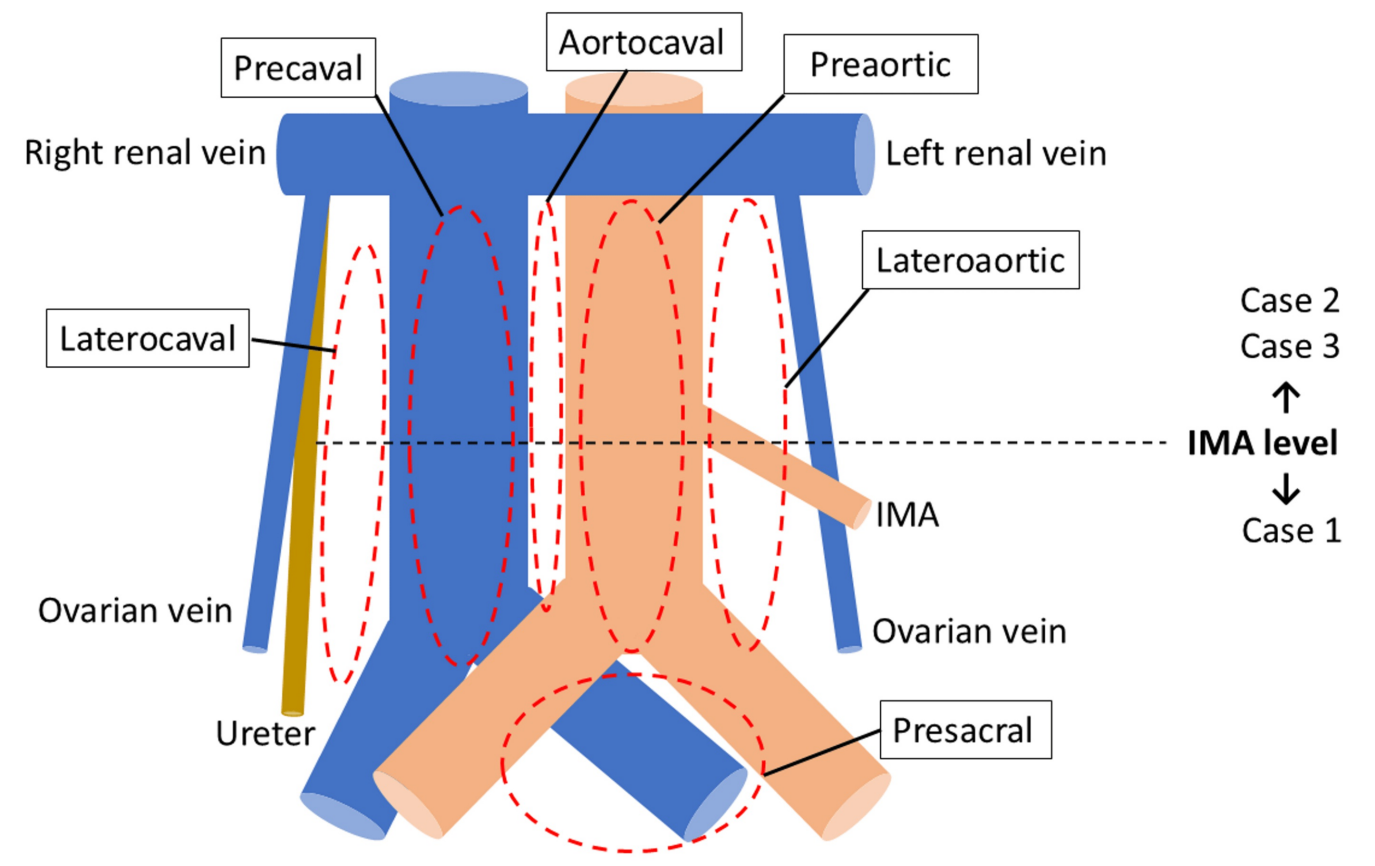
Extent of para-aortic lymphadenectomy. Case 1: Para-aortic lymphadenectomy (PALND) to the level of the inferior mesenteric artery (IMA). Cases 2 and 3: PALND to the level of the left renal vein. Original illustration created by the authors.

PALND was generally performed up to the level of the renal vessels using a transperitoneal approach, as previously reported [11]. However, in technically challenging cases - such as those with severe adhesions, vascular anomalies, or marked obesity - the dissection was limited to the level of the inferior mesenteric artery (Figure 2). Key steps included: (1) incision of peritoneum over the right common iliac artery; (2) creation of peritoneal tent through a suspension of the peritoneum using Laptraction® (Hakko Co., Ltd., Nagano, Japan) [12]; (3) lateral retraction of the right ureter with a silicone sling (Vespasta®; Alfresa Pharma Corporation, Osaka, Japan) [13]; (4) identification of renal vein, psoas muscle, inferior mesenteric artery, gonadal vein, and a left ureter; (5) dissection of nodes from aortic bifurcation to renal veins; and (6) removal of presacral nodes. Specimens were extracted via the 12-mm port or vaginally.

The clinicopathological characteristics and the surgical outcomes of three cases undergoing para-aortic lymphadenectomy using the hinotori™ Surgical Robot System are summarized in Table 1.

**Table 1:** Clinicopathological characteristics of three cases of para-aortic lymphadenectomy using the hinotori™ Surgical Robot System. ^†^Staging of endometrial and ovarian cancers was performed according to the 2009 and 2014 International Federation of Gynecology and Obstetrics classifications, respectively. SH, simple hysterectomy; BSO, bilateral salpingo-oophorectomy; PLND, pelvic lymphadenectomy; PALND, para-aortic lymphadenectomy; OM, omentectomy; AP, adjuvant chemotherapy consisting of doxorubicin plus cisplatin

	Case 1	Case 2	Case 3
Age (year)	72	62	70
Body mass index (kg/m^2^)	18.2	17.5	19.6
Family history of hereditary cancer	No	No	No
Chief complaint	Abnormal uterine bleeding	Abdominal distension	Abnormal uterine bleeding
Cancer type	Endometrial cancer	Ovarian cancer	Endometrial cancer
Histology	Endometrioid adenocarcinoma (grade 2)	Mucinous adenocarcinoma	Endometrioid adenocarcinoma (grade 3)
Preoperative stage	IB	IB	IB
Surgical procedures	SH, BSO, PLND, PALND, OM	PLND, PALND	PLND, PALND, OM
Indication for surgery^†^	Staging for stage IB disease	Staging for stage IB disease	Staging for stage IB disease
Operative time (minutes)	477	461	512
Blood loss (mL)	25	50	10
Total lymph nodes	25	66	46
Pelvic lymph nodes	16	28	20
Para-aortic lymph nodes	9	38	26
Metastatic lymph nodes	0	0	0
Intraoperative/Postoperative complications	No	No	No
Postoperative adjuvant chemotherapy	AP	No adjuvant	AP
Recurrence (Follow-up duration)	No (14 months)	No (6 months)	No (3 months)

Case 1

**Figure 3:**
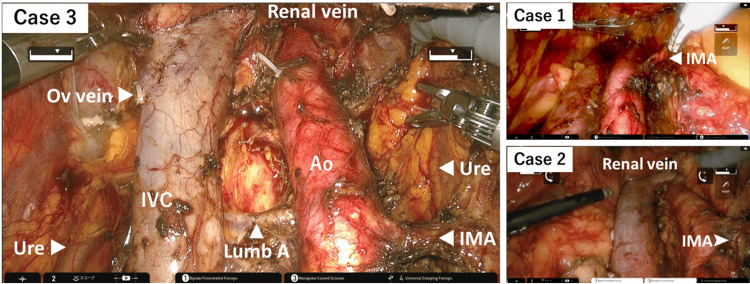
Intraoperative views after completion of para-aortic lymphadenectomy. Representative photographs from three cases. The para-aortic lymphadenectomy included the lateroaortic, preaortic, laterocaval, precaval, aortocaval, and presacral regions, extending from the level of the inferior mesenteric artery (Case 1) or the left renal vein (Cases 2 and 3) to the aortic bifurcation. Ao, descending aorta; IMA, inferior mesenteric artery; IVC, inferior vena cava; Lumb A, lumbar artery; Ov vein, ovarian vein; Ure, ureter

A 72-year-old woman (BMI 18.2 kg/m^2^) with grade 2 endometrioid adenocarcinoma, clinically suspected to be stage IB disease, underwent robotic total hysterectomy, bilateral salpingo-oophorectomy (BSO), pelvic lymphadenectomy (PLND), and PALND up to the inferior mesenteric artery (Figures 2, 3). The operative time was 477 minutes, with an estimated blood loss of 25 mL. Final pathology revealed pT1B N0 M0 disease (grade 2 endometrial carcinoma) with lymphovascular invasion. A total of 25 lymph nodes were retrieved (16 pelvic and 9 para-aortic). She received adjuvant chemotherapy and remained disease-free at 12 months of follow-up.

Case 2

A 62-year-old woman (BMI 17.5 kg/m^2^) with a large mucinous ovarian tumor, initially suspected to be a mucinous borderline tumor, underwent primary open surgery including total hysterectomy, BSO, and omentectomy. However, final pathology confirmed invasive carcinoma. Subsequently, robotic restaging surgery was performed, including PLND and PALND up to the renal vessels (Figures 2, 3). The operative time was 461 minutes, and blood loss was 50 mL. A total of 66 lymph nodes were removed (28 pelvic and 38 para-aortic), all of which were negative. The patient opted for observation without adjuvant therapy and remained recurrence-free at four months.

Case 3

A 70-year-old woman (BMI 19.6 kg/m^2^) with grade 3 endometrial carcinoma, clinically suspected to be stage IB disease, underwent robotic total hysterectomy, BSO, omentectomy, PLND, and PALND up to the renal vessels (Figures 2, 3). The operative time was 512 minutes, with minimal blood loss of 10 mL. Final pathology demonstrated pT1A N0 M0 disease (grade 3 endometrial carcinoma) without lymphovascular invasion. A total of 46 lymph nodes were retrieved (20 pelvic and 26 para-aortic), all negative. She is currently receiving adjuvant chemotherapy.

## Discussion

This study was designed as an exploratory, early-phase investigation to address the question of whether PALND can be technically performed using the hinotori™ Surgical Robot System. Accordingly, we reported a case series comprising the first three patients who underwent hinotori™-assisted PALND at our institution. 

As of 2024, four robotic platforms have been approved in Japan: da Vinci® (2009), Hugo™ (2020), Saroa™ (2022), and hinotori™ (2023). To date, only the da Vinci® system has been reported for use in PALND [14]. Barriers to wider adoption of hinotori™ include its recent approval and the absence of insurance reimbursement for robotic PALND, unlike laparoscopic PALND.

To our knowledge, this is the first study to demonstrate the feasibility and safety of PALND performed using the hinotori™ Surgical Robot System, a domestically developed robotic platform in Japan. These findings suggest that the hinotori™ system may be applicable to advanced gynecologic oncologic procedures beyond endopelvic surgery and could provide an additional option for institutions in Japan where the system is available.

In our three cases, 9-38 para-aortic nodes were retrieved, comparable to or exceeding the 6-33 nodes typically reported for laparoscopic or da Vinci®-assisted PALND [14,15], suggesting an oncologic adequacy. No conversions or intraoperative complications occurred, and blood loss was minimal (10-50 mL), confirming safety. Postoperative recovery was uneventful in all cases, supporting feasibility. Operative times (461-512 minutes) were also consistent with Japanese reports (371-834 minutes, median ~480) [14]. The relatively lengthy operative times reflect the academic training environment, where junior surgeons performed pelvic procedures, including hysterectomy, salpingo-oophorectomy, and pelvic lymphadenectomy, while the most experienced surgeon performed the PALND. Operative times are expected to decrease as surgical proficiency increases.

The limitations of this study should be acknowledged. As this study was designed as a feasibility assessment rather than to validate therapeutic efficacy, this and the previous sections primarily focus on surgical outcomes, including operative time, blood loss, and perioperative complications. The number of retrieved para-aortic lymph nodes, one of the most important indicators of procedural adequacy in PALND, was carefully evaluated and discussed in comparison with previously published reports. However, oncologic outcomes were not assessed because of the short follow-up duration and the limited number of cases. Comparisons with laparoscopic or da Vinci®-assisted PALND were restricted to descriptive references based on values reported in the literature, without statistical analysis or formal validation.

With further accumulation of cases, future studies will be able to address the limitations of the present report and allow for more comprehensive evaluation of procedural safety, reproducibility, and oncological validity.

## Conclusions

PALND using the hinotori™ Surgical Robot System may be technically feasible in selected cases, with acceptable short-term perioperative outcomes. These first reported cases provide preliminary clinical experience regarding the use of hinotori™ in oncologic surgery. Further accumulation of cases is necessary to clarify technical reproducibility and oncologic safety.
